# Screening and Identification of Potential Biomarkers in Hepatitis B Virus-Related Hepatocellular Carcinoma by Bioinformatics Analysis

**DOI:** 10.3389/fgene.2020.555537

**Published:** 2020-09-30

**Authors:** Xian-Chang Zeng, Lu Zhang, Wen-Jun Liao, Lu Ao, Ze-Man Lin, Wen Kang, Wan-Nan Chen, Xu Lin

**Affiliations:** ^1^Key Laboratory of Gastrointestinal Cancer, Ministry of Education, School of Basic Medical Sciences, Fujian Medical University, Fuzhou, China; ^2^Fujian Key Laboratory of Medical Bioinformatics, Department of Bioinformatics, School of Basic Medical Sciences, Fujian Medical University, Fuzhou, China; ^3^Fujian Key Laboratory of Tumor Microbiology, Department of Medical Microbiology, Fujian Medical University, Fuzhou, China

**Keywords:** hepatitis B virus, hepatocellular carcinoma, hub genes, biomarkers, bioinformatics analysis

## Abstract

Hepatocellular carcinoma (HCC) is one of the most lethal cancers globally. Hepatitis B virus (HBV) infection might cause chronic hepatitis and cirrhosis, leading to HCC. To screen prognostic genes and therapeutic targets for HCC by bioinformatics analysis and determine the mechanisms underlying HBV-related HCC, three high-throughput RNA-seq based raw datasets, namely GSE25599, GSE77509, and GSE94660, were obtained from the Gene Expression Omnibus database, and one RNA-seq raw dataset was acquired from The Cancer Genome Atlas (TCGA). Overall, 103 genes were up-regulated and 127 were down-regulated. A protein–protein interaction (PPI) network was established using Cytoscape software, and 12 pivotal genes were selected as hub genes. The 230 differentially expressed genes and 12 hub genes were subjected to functional and pathway enrichment analyses, and the results suggested that cell cycle, nuclear division, mitotic nuclear division, oocyte meiosis, retinol metabolism, and p53 signaling-related pathways play important roles in HBV-related HCC progression. Further, among the 12 hub genes, kinesin family member 11 (KIF11), TPX2 microtubule nucleation factor (TPX2), kinesin family member 20A (KIF20A), and cyclin B2 (CCNB2) were identified as independent prognostic genes by survival analysis and univariate and multivariate Cox regression analysis. These four genes showed higher expression levels in HCC than in normal tissue samples, as identified upon analyses with Oncomine. In addition, in comparison with normal tissues, the expression levels of KIF11, TPX2, KIF20A, and CCNB2 were higher in HBV-related HCC than in HCV-related HCC tissues. In conclusion, our results suggest that KIF11, TPX2, KIF20A, and CCNB2 might be involved in the carcinogenesis and development of HBV-related HCC. They can thus be used as independent prognostic genes and novel biomarkers for the diagnosis of HBV-related HCC and development of pertinent therapeutic strategies.

## Introduction

Liver cancer is the most common type of cancer across the world, accounting for 8.2% of cancer deaths (Bray et al., 2018). Hepatocellular carcinoma (HCC) is the most common primary liver malignancy and the leading cause of liver cancer-related deaths globally (Venook et al., 2010). HCC is difficult to diagnose at an early stage and challenging to treat. It can be caused by several risk agents, such as chronic infection with hepatitis B virus (HBV) or hepatitis C virus (HCV), and exposure to alcohol and aflatoxins (Wang et al., 2002; El-Serag and Rudolph, 2007). In Asian countries, most cases of HCC are associated with chronic HBV infection (Beasley et al., 1981). HCC is associated with high recurrence and drug resistance; thus, it is urgent to identify potential biomarkers during chronic HBV infection to precisely predict HCC progression and to determine better therapeutic targets. HBV-induced HCC involves a complex, gradual process and includes the integration of HBV DNA into host cell DNA (Wang et al., 2017). HBV proteins, including HB× and MHBSt, have oncogenic potential themselves; in addition, some oncogenes in hepatocytes are potentially regulated by HBV proteins via protein-protein interactions, participating in the initiation and progression of HBV-induced HCC (Levrero and Zucman-Rossi, 2016). However, the molecular mechanisms underlying the initiation, progression and metastasis of HBV-induced HCC remain far from being fully understood.

In recent years, the exploration of genes related to carcinogenesis and development of HCC by bioinformatics methods have been increasing. TP53 (Kan et al., 2013), UBE3C (Jiang et al., 2014), SHP-1 (Wen et al., 2018), COL1A1 (Ma et al., 2019), CD5L, and SLC22A10 (Zhang et al., 2019) have been reported to be potential therapeutic targets of HCC by high-throughput sequencing-based bioinformatics analysis. The NCBI Gene Expression Omnibus (GEO) and the Cancer Genome Atlas (TCGA) databases, which provide comprehensive profiles of gene expression data, have been extensively applied to investigate the carcinogenesis of HCC by bioinformatics mining. Further, the potential molecular mechanisms underlying HBV-related HCC can be speculated via hub genes identification by bioinformatics analysis. In the present study, three high-throughput RNA-Seq-based raw datasets from the GEO database and one dataset from TCGA were downloaded, and these included 97 normal, 47 HBV-related HCC, and 374 HCC specimens. We identified 230 differentially expressed genes (DEGs) and 12 hub genes. Among the 12 hub genes, kinesin family member 11 (KIF11), TPX2 microtubule nucleation factor (TPX2), kinesin family member 20A (KIF20A), and cyclin B2 (CCNB2) were found to be independent prognostic markers of HBV-related HCC. We believe that our results should help us better comprehend the mechanisms underlying HBV-related HCC and facilitate the identification of potential targets for the diagnosis and treatment of HCC.

## Materials and Methods

### Raw RNA-seq Dataset Collection

For screening DEGs, three high-throughput RNA-seq-based raw datasets, namely GSE25599 (Huang et al., 2011), GSE94660 (Yoo et al., 2017), and GSE77509 (Yang et al., 2017), which comprised patients with HBV infection, were downloaded from the NCBI GEO database^1^. GSE94660 (21 paired normal and HBV-related HCC tissue samples) and GSE77509 (16 paired normal and HBV-related HCC tissue samples) were established using GPL16791 Illumina HiSeq 2500 (*Homo sapiens*), while GSE25599 (10 paired normal and HBV-related HCC tissue samples) was established using the GPL9052 Illumina Genome Analyzer. RNA-seq raw data and clinical data of 50 normal samples and 374 HCC samples^2^ were downloaded from TCGA^3^. For the validation of independent prognostic genes, a dataset named LIRI-JP^4^, including 202 normal and 243 HCC tissue samples, was downloaded from the International Cancer Genome Consortium (ICGC)^5^. For comparing the expression levels of independent prognostic genes between HCV- and HBV-related HCC, the GSE69715 dataset [66 normal and 37 HCV-related HCC tissue samples established using GLP570 (HG-U133_Plus_2)] was downloaded from the GEO database.

### Data Processing and DEGs Screening

Gene expression profile matrix files of GSE25599, GSE77509, and GSE94660 were obtained from raw datasets using Perl (de Hoon et al., 2004). Nevertheless, the gene expression profile matrix data of TCGA was acquired using the “TCGAbiolinks” R package (Colaprico et al., 2016) and Perl. Genes that were differentially expressed between normal and HBV-related HCC tissue samples were screened by the limma R (Ritchie et al., 2015) and edgeR R packages (Robinson et al., 2010). | Log_2_(FC)| ≥ 1.0, *p*-value ≤ 0.05, and FDR ≤ 0.05 were set as the cutoff criteria for DEGs screening after background correction and data normalization. Overlapped DEGs among GSE25599, GSE77509, GSE94660, and TCGA were identified using the VennDiagram R package (Chen and Boutros, 2011). The heatmaps of DEGs, which could be divided into up- and down-regulated groups, were drawn using the “pheatmap” R package (Galili et al., 2018).

### Gene Ontology (GO) and Kyoto Encyclopedia of Genes and Genomes (KEGG) Pathway Enrichment Analyses

The names of DEGs were translated into gene IDs using the R programming language. To investigate the biological pathways that might be involved in the occurrence and development of HBV infection and HCC, candidate DEGs were segregated into up- and down-regulated groups and subjected to pathway enrichment analysis. Gene Ontology (GO) analysis, which involved three categories, namely molecular functions (MF), cellular components (CC), and biological processes (BP), and Kyoto Encyclopedia of Genes and Genomes (KEGG) pathway enrichment analysis were performed with the threshold of FDR-value < 0.05 using the clusterProfiler R package (Yu et al., 2012), which facilitated biological terminology classification and gene cluster enrichment.

### Protein–Protein Interaction (PPI) Network Analysis and Hub Gene Screening

A protein–protein interaction (PPI) network of DEGs was constructed using STRING^6^ (Szklarczyk et al., 2019) and visualized with Cytoscape v3.6.1 (Shannon et al., 2003). DEGs that consisted of several important nodes with many other interaction partners were analyzed using Molecular Complex Detection (Bader and Hogue, 2003) and CytoHubba (Chin et al., 2014; Tang et al., 2019). Subnets of the vast protein interaction network were extracted by calculating the degree of nodes, and highly connected nodes with a degree score of >45 and *p*-value < 0.05 were identified as hub genes.

### Survival Analysis of Hub Genes

Survival analysis were primarily performed using clinical data from TCGA to predict the prognostic value of hub genes. Kaplan–Meier survival curves of hub genes were plotted using the survival R package^7^ and differences in survival rate were evaluated with a log-rank test threshold of *p*-value < 0.05. To evaluate the accuracy of the survival curves, receiver operating characteristic (ROC) curves were then constructed using the “survival ROC” R package (Huang et al., 2017) with the threshold of AUC ≥ 0.6. Next, Cox proportional-hazards models were used to estimate the effects of prognostic factors on survival using the survival R and “survminer” R packages^8^ with the threshold of *p*-value < 0.05. Univariate Cox analysis was first performed to screen for genes significantly associated with overall survival rate, and multivariate Cox analysis was then performed to identify independent prognostic genes (Orimo et al., 2008; Uemura et al., 2009).

### Expression Analysis of Independent Prognostic Genes for HCC Using TCGA Dataset

To validate independent prognostic genes for HCC screened by survival analyses, the aforementioned TCGA clinical data were used to analyze individual gene expression levels between normal and HCC tissue specimens at different stages of tumor progression using the “ggpubr” R package^9^. Data pertaining to normal and HCC tissue samples were compared using Wilcoxon test, and those pertinent to multiple samples from different stages of tumor progression were compared using the Kruskal–Wallis test, with the threshold of *p*-value < 0.05.

### Validation of Potential Prognostic Biomarkers in HCC Using a Dataset From the International Cancer Genome Consortium (ICGC)

To further evaluate the clinical value of the independent prognostic genes, a dataset of patients with HCC was downloaded from the ICGC portal (see text footnote 4) for survival and ROC curve analyses; for this purpose, we used the survival R package, survival ROC R package, and Perl. Ultimately, a meta-analysis of the independent prognostic genes in Oncomine^10^ (Rhodes et al., 2004), a cancer-profiling database containing published data and listing differential gene expression analyses, were performed to verify their expression levels in patients with HCC using four published data (Chen et al., 2002; Wurmbach et al., 2007; Roessler et al., 2010).

### Correlation Analysis of Potential Prognostic Biomarkers in HCC

To analyze the potential relationships among the four independent prognostic genes in HCC occurrence and development, TCGA dataset was subjected to correlation analyses using the corrplot^11^ R software. The correlation coefficient (Cor), ranging from −1 (perfect negative correlation) to +1 (perfect positive correlation), indicated how closely data in a scatterplot were arranged along a straight line. *p*-value < 0.05 for the coefficients indicates a statistically significant relationship.

### Expression Levels of Potential Prognostic Biomarkers in HCV- and HBV-Related HCC

Since viral Hepatitis B and Hepatitis C are the most commonly implicated risk factors for HCC, to compare the expression levels of the independent prognostic genes between HCV-related HCC and HBV-related HCC, the GSE69715 dataset for HCV and the GSE94660 dataset for HBV were analyzed using gglpot2^12^, cowplot^13^, and ggpubr^14^ package. The relative expression levels (i.e., fold change) of these four genes in the tumor tissues comparing with normal tissues were calculated. Wilcoxon test was carried out between the HCC and normal tissues. *p*-value < 0.05 indicate statistical significance.

## Results

### Identification of DEGs

**FIGURE 1:**
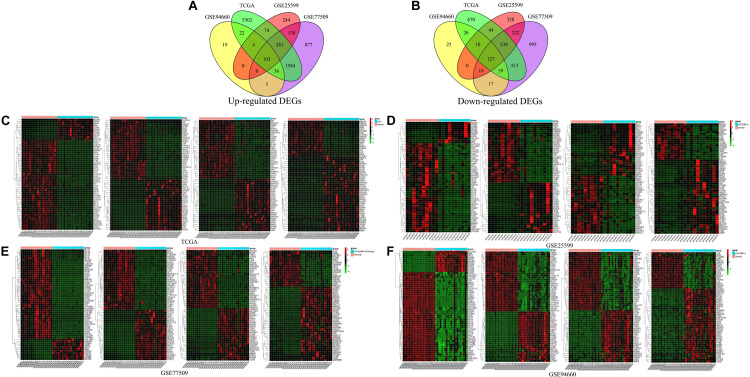
Venn diagram and cluster heatmap of differentially expressed genes. **(A,B)** Venn diagram showing overlapping DEGs. DEGs were screened based on the following criteria: | Log_2_(FC)| ≥ 1.0, *p*-value ≤ 0.05, and FDR ≤ 0.05. In total, 230 DEGs were overlapping among the GSE25599, GSE94660, GSE77509, and TCGA datasets; of these, 103 genes were up-regulated and 127 were down-regulated. **(C–F)** Cluster heatmaps of DEGs in the four datasets. Colors indicate gene expression levels. Red represents up-regulated genes, and green represents down-regulated genes. For GSE25599, GSE94660, GSE77509 datasets, all of the samples are shown in the heatmaps. However, for TCGA dataset, 46 samples (23 normal and 23 HCC tissue samples) were randomly selected from 424 samples for display convenience.

Differentially expressed genes were identified from three raw datasets, namely GSE25599, GSE94660, and GSE77509, downloaded from the NCBI GEO database and one downloaded dataset from TCGA database using the limma R package and edgeR R package. The cutoff criteria were | log_2_(FC)| ≥ 1.0, *p*-value ≤ 0.05 and FDR ≤ 0.05. In total, 230 DEGs were overlapping among the four datasets, of which 103 were up-regulated (Figure 1A) and 127 were down-regulated (Figure 1B). Cluster heatmaps showing the expression levels of the 230 DEGs in each of the four datasets were generated (Figures 1C–F). Details of the top 20 up- and 20 down-regulated DEGs in HBV-related HCC are shown in Supplementary Table S1.

### Pathway Enrichment Analysis of DEGs

**FIGURE 2:**
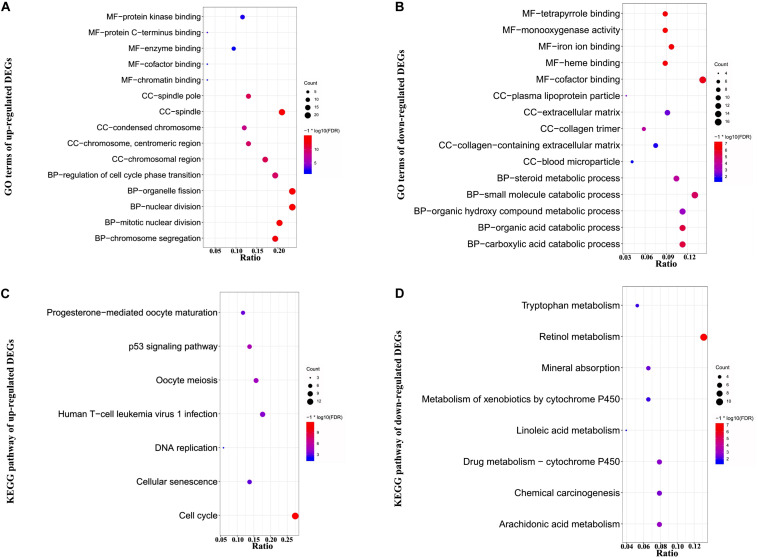
Functional GO and KEGG pathway enrichment analysis of DEGs. DEGs were subjected to GO and KEGG pathway enrichment analyses with the criteria of FDR <0.05. **(A,B)** Plots of significantly enriched GO terms of up- and down-regulated genes for molecular function (MF), cellular components (CC), and biological processes (BP). **(C,D)** Significantly enriched KEGG pathway terms of up- and down-regulated genes. The *y*-axis shows GO category or KEGG pathway, and the *x*-axis shows gene ratio for each individual category. Count represents the number of genes enriched in the corresponding category. –log10 (FDR) represents the logarithm of adjusted *p*-value. The smaller the FDR, the deeper the red color; and the larger the FDR, the deeper the blue color.

To investigate the functional annotation of DEGs, GO, and KEGG pathway enrichment analyses were performed. The results were considered to be statistically significant if FDR value was <0.05. The top 15 GO terms of up-regulated genes are listed in Supplementary Table S2. As evident from Figure 2A and Supplementary Table S2, in the MF, CC, and BP categories, the up-regulated genes were significantly enriched in nuclear division, organelle fission, and mitotic nuclear division; spindle, chromosomal region, and spindle pole; and protein kinase binding, enzyme binding, and chromatin binding, respectively. Further, KEGG pathway analysis of the up-regulated genes indicated that they were primarily enriched in cell cycle, p53 signaling pathway, and oocyte meiosis (Figure 2C and Supplementary Table S3).

As evident from Figure 2B,D and Supplementary Tables S4, S5, in the MF, CC, and BP categories, the down-regulated genes were mainly involved in small molecule catabolic process, organic acid catabolic process, carboxylic acid catabolic process, extracellular matrix, collagen-containing extracellular matrix, collagen trimer, cofactor binding, iron ion binding, and monooxygenase activity, respectively. Moreover, KEGG pathway analysis of the down-regulated genes indicated that they were enriched in retinol metabolism, arachidonic acid metabolism, and drug metabolism–cytochrome P450.

### PPI Network Construction of DEGs and Identification of Hub Genes

**FIGURE 3:**
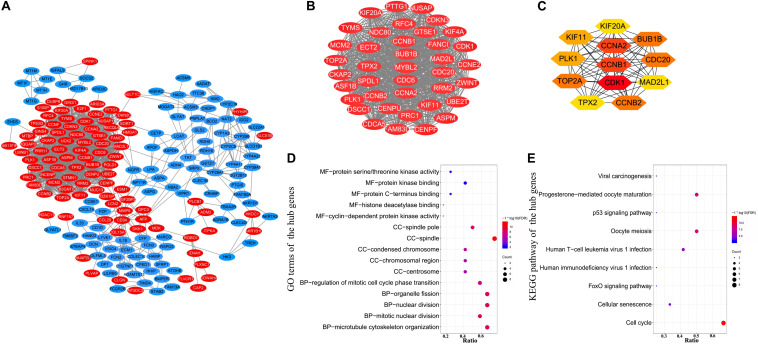
Protein–protein interaction network construction and hub genes identification. **(A)** PPI network of 230 DEGs was visualized using Cytoscape. Red color represents up-regulated genes, and blue color represents down-regulated genes. **(B)** A subnet with 39 nodes and 698 edges was extracted from the PPI network using Molecular Complex Detection and CytoHubba based on the following criteria: degree score >30 and *p*-value < 0.05. **(C)** Hub genes and their co-expression network. Twelve pivotal genes were identified as hub genes using CytoHubba, according to degree score >45 and *p*-value < 0.05. **(D)** GO enrichment analysis of the 12 hub genes. **(E)** KEGG pathway enrichment analysis of the 12 hub genes.

A PPI network of DEGs (Figure 3A) containing 230 nodes and 1189 edges was constructed by STRING and visualized by Cytoscape, which provides critical assessment and integration of protein-protein interactions, including direct (physical) and indirect (functional) correlations. Pivotal modules of the network were obtained using Molecular Complex Detection, and the degree of nodes was calculated using CytoHubba. In the PPI network, the number of edges involved determines the degree score of nodes; the nodes with high degree scores were considered to be hub genes (Chin et al., 2014). 54 DEGs with a degree score of >10 and *p*-value < 0.05 are listed in Supplementary Figure S1. There were 39 genes with degree scores of >30, 24 genes with degree scores of >40, 12 genes with degree scores of >45, and only one gene with degree score of >50, and all of these genes meet the requirements of *p*-value < 0.05. The modules with 39 nodes and 698 edges (degree score >30 and *p*-value < 0.05) were extracted to construct a subnet (Figure 3B). The most significant modules of 12 genes (degree score >45 and *p*-value < 0.05; Figure 3C) were identified as hub genes. The names, abbreviations, and scores of hub genes are summarized in Supplementary Table S6. The top five hub genes with the highest interaction node degrees were CDK1, CCNB1, CCNA2, BUB1B, and CCNB2, implying their potential roles in the development of HBV-related HCC.

Gene Ontology and KEGG pathway enrichment analyses were utilized to investigate the functional enrichment of the 12 hub genes. In the MF, CC, and BP categories, these 12 hub genes were mainly enriched in nuclear division, organelle fission, mitotic nuclear division, spindle, spindle pole, condensed chromosome, protein kinase binding, protein C-terminus binding, and protein serine/threonine kinase activity, respectively (Figure 3D and Supplementary Table S7). Further, KEGG pathway analysis for the hub genes (Figure 3E and Supplementary Table S8) indicated that they were primarily enriched in cell cycle, progesterone-mediated oocyte maturation, and oocyte meiosis.

### Survival Analysis of Hub Genes

**FIGURE 4:**
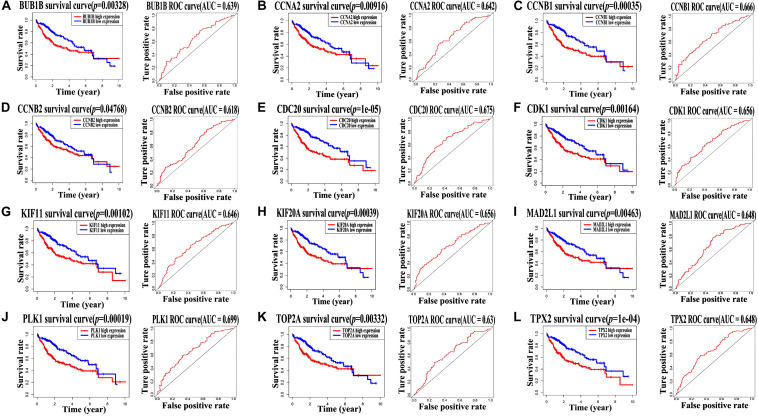
TCGA dataset analysis showed all of the 12 hub genes are related to worse survival rate. Survival analysis of the 12 hub genes, including **(A)** BUB1B, **(B)** CCNA2, **(C)** CCNB1, **(D)** CCNB2, **(E)** CDC20, **(F)** CDK1, **(G)** KIF11, **(H)** KIF20A, **(I)** MAD2L1, **(J)** PLK1, **(K)** TOP2A, and **(L)** TPX2, was performed using Kaplan–Meier survival curves and ROC curves based on clinical data from TCGA dataset. Log-rank test *p*-value < 0.05 and AUC ≥ 0.6 indicate a statistically significant difference.

It is noteworthy that all the 12 hub genes were up-regulated in patients with HBV-related HCC. To explore their prognostic importance, all of them were evaluated using the Kaplan–Meier plot and ROC curve with clinical and expression data from TCGA. As shown in Figure 4, based on their expression levels, AUC values of the 12 hub genes (BUB1B, CCNA2, CCNB1, CCNB2, CDC20, CDK1, KIF11, KIF20A, MAD2L1, PLK1, TOP2A, and TPX2), ranged from 0.6 to 0.7, while log-rank test showed *p*-value < 0.05 in all of the survival curves. Therefore, we considered that all the 12 hub genes appeared to be capable of survival prediction with the thresholds of *p*-value < 0.05 and AUC ≥ 0.6. Patients with HCC and up-regulation of these genes showed worse survival rate.

Further, univariate and multivariate Cox regression analyses were performed to analyze their independent prognostic importance in patients with HCC. As indicated in Table 1, univariate Cox regression analysis showed that all the 12 hub genes were high-risk genes (hazard ratio >1, *p*-value < 0.05); however, multivariate Cox regression analysis suggested that only KIF11, TPX2, KIF20A, and CCNB2 were independent prognostic genes in case of patients with HCC (hazard ratio >1, *p*-value < 0.05).

**TABLE 1:** Univariate and Multivariate COX analysis of hub genes.

**Univariate Cox analysis**					**Multivariate Cox analysis**				
**Gene**	**HR**	**HR.95L**	**HR.95H**	***p*-value**	**Gene**	**HR**	**HR.95L**	**HR.95H**	***p*-value**
BUB1B	1.000874808	1.000446406	1.001303393	6.25E-05	BUB1B	1.00006296	0.998838802	1.001288621	0.919751733
CCNB1	1.000422356	1.00026182	1.000582918	2.15E-07	CCNB1	0.99987269	0.999416831	1.000328765	0.584250641
CCNA2	1.000071977	0.999965794	1.000178172	0.183996138	CCNA2	1.00003697	0.999894372	1.000179596	0.611349315
CDC20	1.000309776	1.000189157	1.000430411	4.80E-07	CDC20	1.000161	0.999873377	1.000448709	0.272621039
CDK1	1.000378834	1.000185759	1.000571947	0.000120059	CDK1	1.00055335	0.999944421	1.001162648	0.074908843
KIF11	1.000641363	1.000273012	1.001009849	6.42E-04	KIF11	0.99882365	0.997724432	0.999924087	0.036162815
KIF20A	1.001021556	1.000695409	1.00134781	8.19E-10	KIF20A	1.0013795	1.000549474	1.002210223	0.001120655
MAD2LA	1.000530847	1.000204046	1.00105783	3.76E-03	MAD2LA	0.99979487	0.998893726	1.000696819	0.655658215
PLK1	1.000116506	1.000414122	1.000955415	7.04E-07	PLK1	1.00043079	0.999673949	1.001188201	0.264671652
T0P2A	1.000641889	1.000049283	1.000183733	0.000681365	TOP2A	0.99987989	0.999683667	1.00007614	0.230289833
TPX2	1.000313957	1.000196027	1.000431901	1.83E-07	TPX2	1.00047491	1.000131646	1.000818286	0.006691495
CCNB2	1.000580374	1.000212605	1.000948277	1.98E-03	CCNB2	0.9981437	0.997060681	0.999227895	0.000795237

### Validation of Potential Prognostic Biomarkers

**FIGURE 5:**
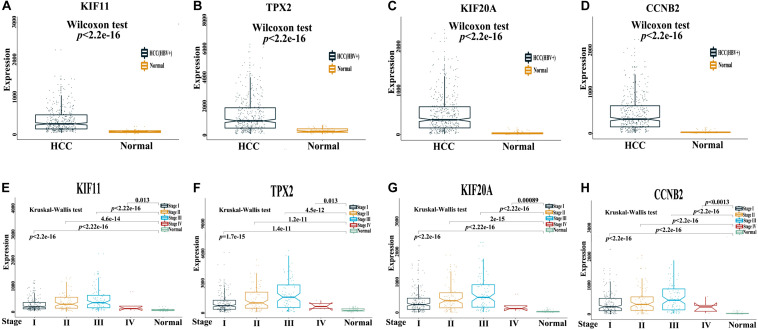
mRNA expression levels of four independent prognostic genes in the HCC tissue samples. TCGA dataset of paired normal and HCC tissue samples **(A–D)** and multiple samples from different stages (I–IV) of HCC progression **(E–H)** were used to investigate mRNA expression levels of four independent prognostic genes, namely KIF11, TPX2, KIF20A, and CCNB2, in patients with HCC. A *p*-value less than 0.05 is statistically significant.

The Cancer Genome Atlas dataset of normal and HCC tissue samples were subjected to Wilcoxon test; KIF11, TPX2, KIF20A, and CCNB2 were found to have higher mRNA expression levels in HCC than in normal tissue samples (Figures 5A–D). Furthermore, the expression levels of KIF11, TPX2, KIF20A, and CCNB2 in multiple samples from different stages (I–IV) of tumor progression were compared using the Kruskal–Wallis test, and the results revealed that in comparison with normal tissue samples, the expression levels of KIF11, TPX2, KIF20A, and CCNB2 were higher at each stage of HCC (Figures 5E–H). These findings indicated the potential roles of these genes for diagnostic and prognostic prediction.

**FIGURE 6:**
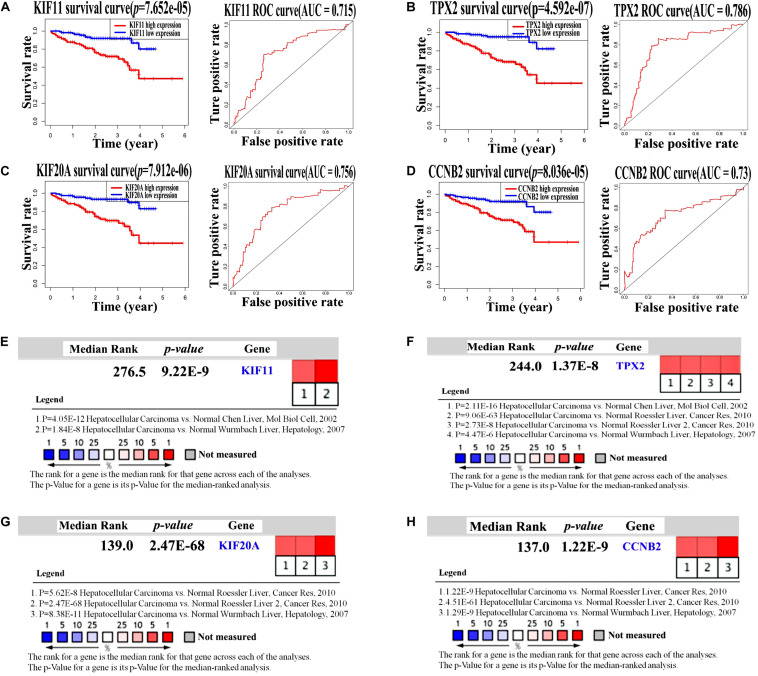
Validation of four independent prognostic genes in the ICGC dataset by survival curves and Oncomine expression analysis. **(A–D)** Kaplan–Meier survival curves and ROC curves of KIF11, TPX2, KIF20A, and CCNB2 genes in the HCC patients from the ICGC dataset. Log-rank test *p*-value < 0.05 and AUC ≥ 0.6 indicate a statistically significant difference. **(E–H)** Oncomine analysis of mRNA expression levels of KIF11, TPX2, KIF20A, and CCNB2 in HCC tissue samples compared with those in normal tissue samples using four sets of published data (*p*-value < 0.05).

Another dataset with RNA-Seq mRNA expression data and clinical pathological data were obtained from the ICGC portal as an independent validation cohort to verify the prognostic potential of KIF11, TPX2, KIF20A, and CCNB2 in HBV-related HCC. Overall survival rate analysis of these four genes was performed using the Kaplan–Meier plot and ROC curves. The results were consistent with those obtained from TCGA datasets, revealing that patients with up-regulated KIF11, TPX2, KIF20A, and CCNB2 genes showed worse survival rate (Figures 6A–D, *p*-value < 0.05 and AUC ≥ 0.6). Notably, as indicated in Figure 6, AUC values calculated using ICGC data were a little higher (from 0.7 to 0.8) compared with those using the TCGA data. In general, an AUC of 0.7 to 0.8 is considered to be acceptable (Mandrekar, 2010). In addition, meta-analysis in Oncomine showed that KIF11, TPX2, KIF20A, and CCNB2 were highly expressed in HCC comparing with normal tissues samples (Figure 6E–H, *p*-value < 0.05). Correlation analyses (Supplementary Figure S2) revealed the potential relationships among these four independent prognostic genes, implying that these four genes have combined effects in HCC occurrence and development.

### Potential Prognostic Biomarkers Showed Lower Relative Expression Levels in HCV-Related HCC Than in HBV-Related HCC

**FIGURE 7:**
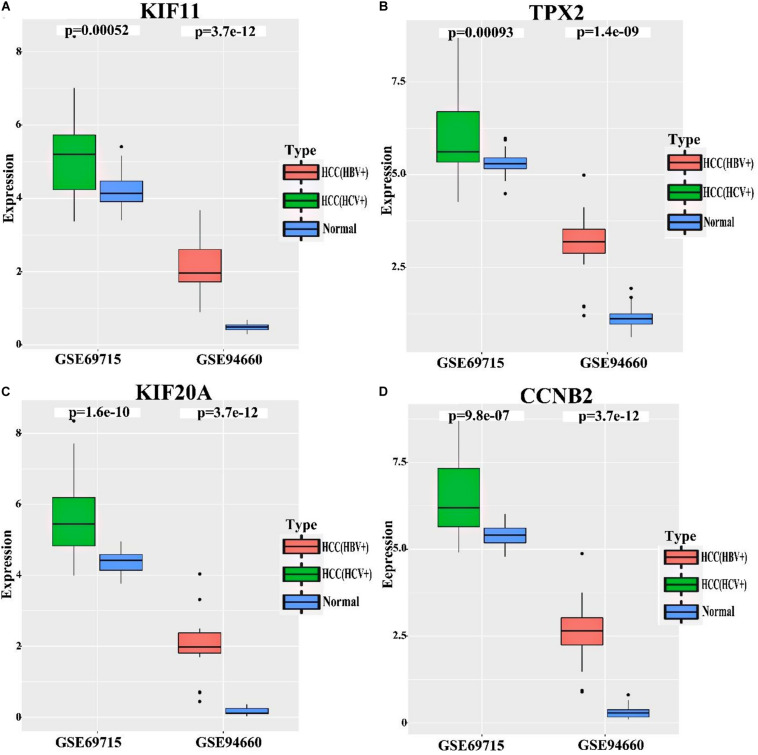
Relative mRNA expression levels of the independent prognostic genes in the HCV-related HCC and HBV-related HCC. Datasets of HCV-related HCC (GSE69715) and HBV-related HCC (GSE94660) were used to analyze relative mRNA expression levels of KIF11 **(A)**, TPX2 **(B)**, KIF20A **(C)**, and CCNB2 **(D)** genes in the tumor tissues comparing with normal tissues. *p*-values < 0.05 indicate statistical significance. The expression level of these four genes in HCV-related HCC, however, only showed a minor increase as compared with that of those in HBV-related HCC.

To deternmine whether KIF11, TPX2, KIF20A, and CCNB2 are specific to HBV-induced HCC comparing with HCV-induced HCC, HCV-related HCC (GSE69715) and HBV-related HCC (GSE94660) datasets were used to analyze the relative expression levels (i.e., fold change) of these 4 genes in HCC and normal tissue samples. As indicated in Figure 7 and Table 2, log_2_(FC) of KIF11, TPX2, KIF20A, and CCNB2 in the HBV-related HCC dataset ranges from 1.38 to 2.66, while log_2_(FC) of these genes in the HCV-related HCC dataset ranges from 0.09 to 0.35. Although all of the *p*-values in the Figure 7 are less than 0.05 (*p*-value < 0.05), which are statistically significant, we do not believe that they are biologically significant, because the expression level of these four genes in HCV-related HCC showed only a minor increase as compared with that in HBV-related HCC.

**TABLE 2:** Compare four independent prognostic genes expression in the HCV-related HCC and HBV-related HCC.

	**Log_2_(FC)**		***p*-value**	
**Gene**	**HBV-HCC**	**HCV-HCC**	**HBV-HCC**	**HCV-HCC**
CCNB2	2.666359727	0.098574137	3.07E-12	9.80E-07
TPX2	1.38249853	0.154570151	1.40E-09	0.00093
KIF20A	2.91389186	0.354370973	3.70E-12	1.60E-10
KIF11	1.875739772	0.275842814	3.70E-12	0.00052

## Discussion

Hepatocellular carcinoma is the most common malignant tumor of the liver. The etiological factors of HCC include hepatitis B or C, aflatoxin, alcohol, and metabolic disorders. In HBV endemic areas, chronic hepatitis B infection has been verified to be closely associated with HCC carcinogenesis (Lavanchy, 2004). Identifying potential biomarkers and elucidating molecular mechanisms of HCC progression are pivotal. Some researchers have used comprehensive bioinformatics analysis to identify hub genes from PPI networks constructed with DEGs, such as for colorectal cancer (Guo et al., 2017), breast cancer (Yang et al., 2019), and non-small-cell lung cancer (Ma et al., 2019). Notably, with bioinformatics analysis, different research groups may identify the same prognostic biomarkers using different datasets (Yang et al., 2019; Nakamura et al., 2020), which may strengthen the significance of data mining. Hepatocarcinogenesis is a complex multifactorial process; in recent decades, bioinformatics analyses of high-throughput data obtained upon using methods such as microarray and new generation sequencing have become common for exploring the mechanisms underlying HCC.

Gene Expression Omnibus is a public functional genomics database and includes a large repository of high-throughput, next-generation sequencing results and related information for over 200 organisms (Barrett et al., 2007). In the present study, to investigate pivotal genes associated with the HBV-related HCC, we used three high-throughput RNA-seq-based datasets, namely GSE25599, GSE94660, and GSE77509, downloaded from GEO, and information pertaining to 47 normal and 47 HBV-related HCC tissue samples was used. At the same time, to reduce the number of DEGs identified and improve accuracy, high-throughput RNA -seq results and clinical information of 50 normal and 374 HCC tissue samples were downloaded from TCGA and used for screening DEGs overlapping with GEO datasets and for survival analysis. In addition, the HCC expression data in the ICGC database were used to validate potential prognostic biomarkers in HCC.

A total of 230 DEGs were identified, of which 127 were down-regulated and 103 were up-regulated, and a PPI network was then constructed. Twelve hub genes – BUBIB, CCNA2, CCNB1, CDC20, CDK1, KIF11, KIF20A, PLK1, TOP2A, MAD2L1, TOP2A, and TPX2 – were identified according to a degree score of >45. GO and KEGG pathway enrichment analyses of the 230 DEGs and 12 hub genes suggested that HBV-related HCC occurrence and development are associated with cell cycle, nuclear division, mitosis, p53 pathway, oocyte meiosis, retinol metabolism, and organic acid catabolism. Cell cycle abnormality evidently has a key role during the process of liver cancer (Choi et al., 2001), and cyclin D1 degradation has been reported to inhibit HCC occurrence (Wu et al., 2018). Further, p53, the most common abnormality of dominant oncogenes in human tumors including HCC (Wu et al., 2018), plays a critical role in cell cycle arrest and apoptosis in response to DNA damage (Khemlina et al., 2017). Alterations in retinol metabolism play a pivotal role in the process of liver fibrosis, and enzymes involved in retinol metabolism are reportedly related to liver cancer (Pettinelli et al., 2018).

To analyze the prognosis and clinical significances of the 12 hub genes in HBV-related HCC, clinical data from TCGA were used for survival and ROC curve analyses. It was found that patients in whom the expression levels of the 12 hub genes were up-regulated showed worse survival rate, indicating their prognostic value for HCC. To further analyze the prognostic value of these genes, univariate and multivariate Cox regression analyses were performed using the 12 hub genes and found that KIF11, TPX2, KIF20A, and CCNB2 might be independent prognostic genes and potential targets for the diagnosis of HBV-related HCC. In addition, it was demonstrated that the expression levels of these four genes were higher in HCC than in normal tissue samples, and their expression levels were also higher at different stages of HCC than those in normal tissue samples. Data pertaining to patients with HCC from the ICGC database further validated that KIF11, TPX2, KIF20A, and CCNB2 were associated with worse survival rates in patients with higher gene expression levels. Oncomine analysis demonstrated that the expression levels of these genes were still higher in different patients with HCC.

Through continuous data filtering using different procedures and different sources of data, the number of candidate genes reduced, making our results more credible. Besides, correlation analyses of KIF11, TPX2, KIF20A, and CCNB2 indicated the potential relationships among them, and suggested that they together promote the occurrence and development of HCC.

The activation of KIF20A–Gli2 axis has been reported to be crucial for hepatoma cell growth, indicating that KIF20A plays a vital role in the development of liver cancer (Shi et al., 2016). Further, an increase in the mRNA expression level of KIF20A and its product MKLP2 has been related to HCC invasion (Gasnereau et al., 2012). KIF11 is related with the progression and prognosis of liver cancer, and its overexpression has been related to low survival rate of patients with liver cancer (Chen et al., 2017). A study reported that CCNB2 overexpression induces the expression of karyopherin subunit-α-2, promoting the cell cycle of HCC cells (Gao et al., 2018). Other studies have reported that the positive regulatory network of CCNB2 is involved in ubiquitination, DNA repair, and cell proliferation in non-tumor hepatitis or cirrhosis induced by HBV (Wang et al., 2012), suggesting that CCNB2 plays a role in HBV-related diseases. Moreover, it was observed that knocking down TPX2 in hepatocarcinoma cell lines effectively reduced cell growth via G2/M blockage and induced apoptosis (Hsu et al., 2017). TPX2 has also been reported to promote HCC development by activating PI3K/Akt signal (Huang et al., 2019).

Datasets of HCV-related HCC and HBV-related HCC were used to compare the expression levels of these four genes between HCV- and HBV-induced HCC. HCV-related HCC showed only a minor increase in the expression levels as compared with HBV-related HCC, indicating that KIF11, TPX2, KIF20A, and CCNB2 might be specific to HBV-induced HCC. But the underlying mechanisms how these four genes may induce the HBV-related HCC need to be further elucidated.

In conclusion, KIF11, TPX2, KIF20A, and CCNB2 seem to play a key role in HBV-related HCC. However, further studies are warranted to explore the mutual influence of these genes and HBV on HBV-related HCC carcinogenesis. Further studies should also identify whether these four genes are induced by factors other than HBV infection in patients with HCC and whether HBV infection itself causes aberrant expression of these genes and promotes HCC progression. Whether HBV-encoded proteins, such as HBV X protein, can interact with intracellular proteins via these four genes and lead to HCC remains to be elucidated.

## Conclusion

Our findings suggest that KIF11, TPX2, KIF20A, and CCNB2 are involved in the carcinogenesis and development of HBV-related HCC. Thus, they can be used as independent prognostic genes for patients with HBV-related HCC and also as novel biomarkers for the diagnosis of HBV-related HCC and development of pertinent therapeutic strategies.

## Data Availability Statement

The data analyzed in this manuscript can be downloaded from the NCBI Gene Expression Omnibus database (http://www.ncbi.nlm.nih.gov/geo) using the accession numbers GSE25599, GSE94660, GSE77509, and GSE69715, and TCGA (https://cancergenome.nih.gov/), and the ICGC (https://dcc.icgc.org/releases/current/Projects/LIRI-JP) database.

## Author Contributions

W-NC and XL conceived and designed the investigation. X-CZ and LZ analyzed the data and drafted the manuscript. W-JL, LA, Z-ML, and WK conducted statistical analyses. All authors have read and approved the manuscript.

## Conflict of Interest

The authors declare that the research was conducted in the absence of any commercial or financial relationships that could be construed as a potential conflict of interest.

## Footnotes

1 http://www.ncbi.nlm.nih.gov/geo

2 https://portal.gdc.cancer.gov/repository

3 https://portal.gdc.cancer.gov/

4 https://dcc.icgc.org/projects/LIRI-JP

5 https://dcc.icgc.org/releases/current/Projects

6 http://string-db.org/

7 https://CRAN.R-project.org/view=Survival

8 https://CRAN.R-project.org/package=survminer

9 https://CRAN.R-project.org/package=ggpubr

10 https://www.oncomine.org/resource/login.html

11 https://github.com/taiyun/corrplot

12 https://github.com/tidyverse/ggplot2

13 https://github.com/wilkelab/cowplot/issues

14 https://rpkgs.datanovia.com/ggpubr/

## Supplementary Material

FIGURE S1 DEGs with degree scores of >10 in the PPI network. Fifty-four DEGs showed a degree score of >10 (arranged from largest to smallest).

FIGURE S2 Correlation among KIF11, TPX2, KIF20A, and CCNB2 genes in HCC occurrence and development. Correlation analysis of KIF11, TPX2, KIF20A, and CCNB2 in HCC occurrence and development showed the potential relationships among these independent prognostic genes. Correlation coefficient (Cor) represents the correlation coefficient. *p*-value < 0.05 indicates a statistically significant difference.

TABLE S1 The Top 20 up-regulated and 20 down-regulated DEGs between HCC and Normal tissues.

TABLE S2 The Top 15 significantly enriched GO terms of the 103 up-regulated DEGs.

TABLE S3 Enriched KEGG pathyways of the 103 up-regulated DEGs.

TABLE S4 The Top 15 significantly enriched GO terms of the 127 down-regulated DEGs.

TABLE S5 Enriched KEGG pathyways of the 127 down-regulatde DEGs.

TABLE S6 Degree scores of the hub genes.

TABLE S7 The Top 15 significantly enriched GO terms of the hub genes.

TABLE S8 Enriched KEGG pathyways of the hub genes.
