# Supplementary figures and images for: Screening and Identification of Potential Biomarkers in Hepatitis B Virus-Related Hepatocellular Carcinoma by Bioinformatics Analysis
Source: Front Genet. 2020 Sep 30;11:555537. doi: 10.3389/fgene.2020.555537 (PMC7556301; doi:10.3389/fgene.2020.555537)

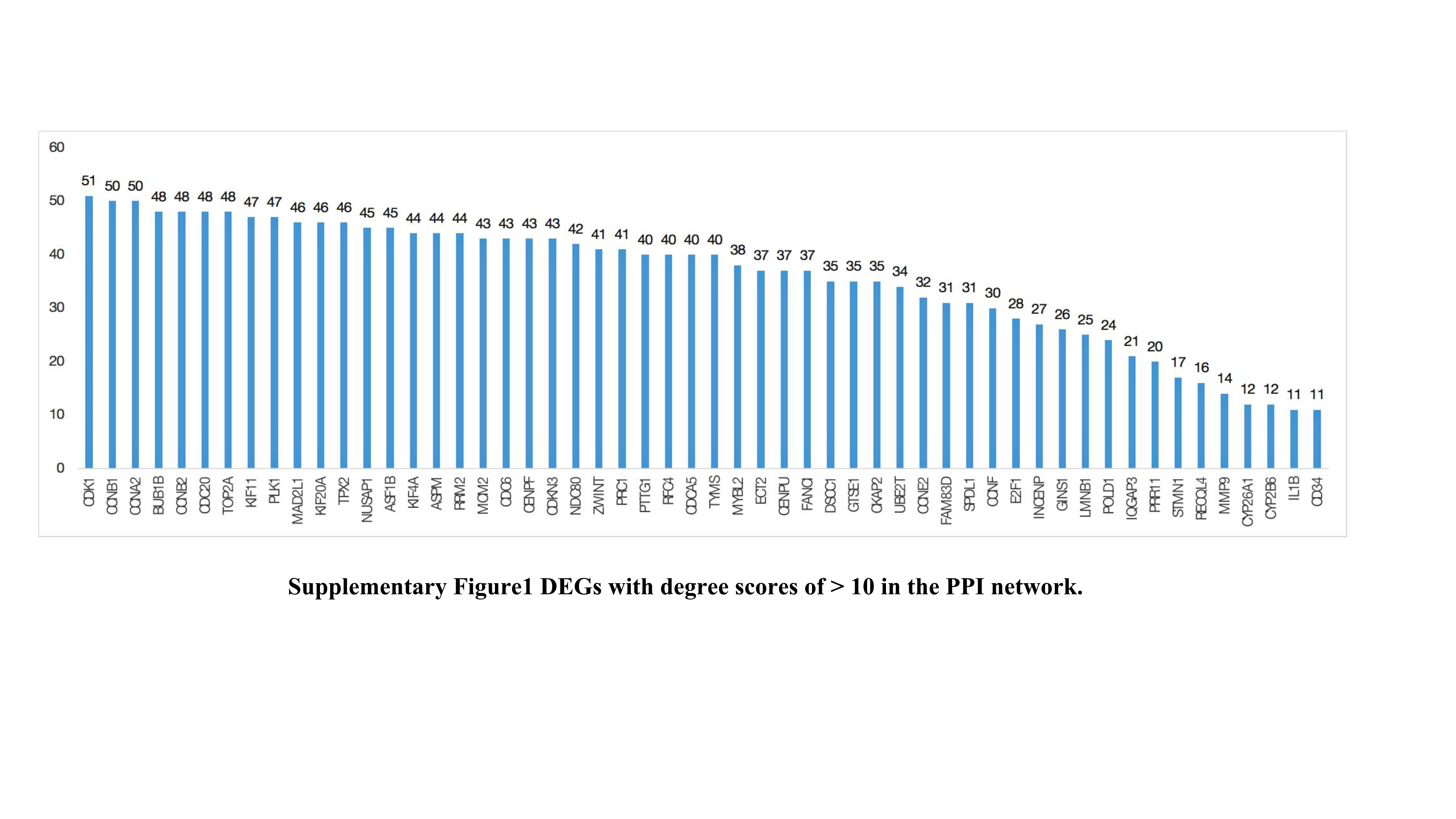

Supplement: FIGURE S1 — DEGs with degree scores of >10 in the PPI network. Fifty-four DEGs showed a degree score of >10 (arranged from largest to smallest). [file Image_1.JPEG]

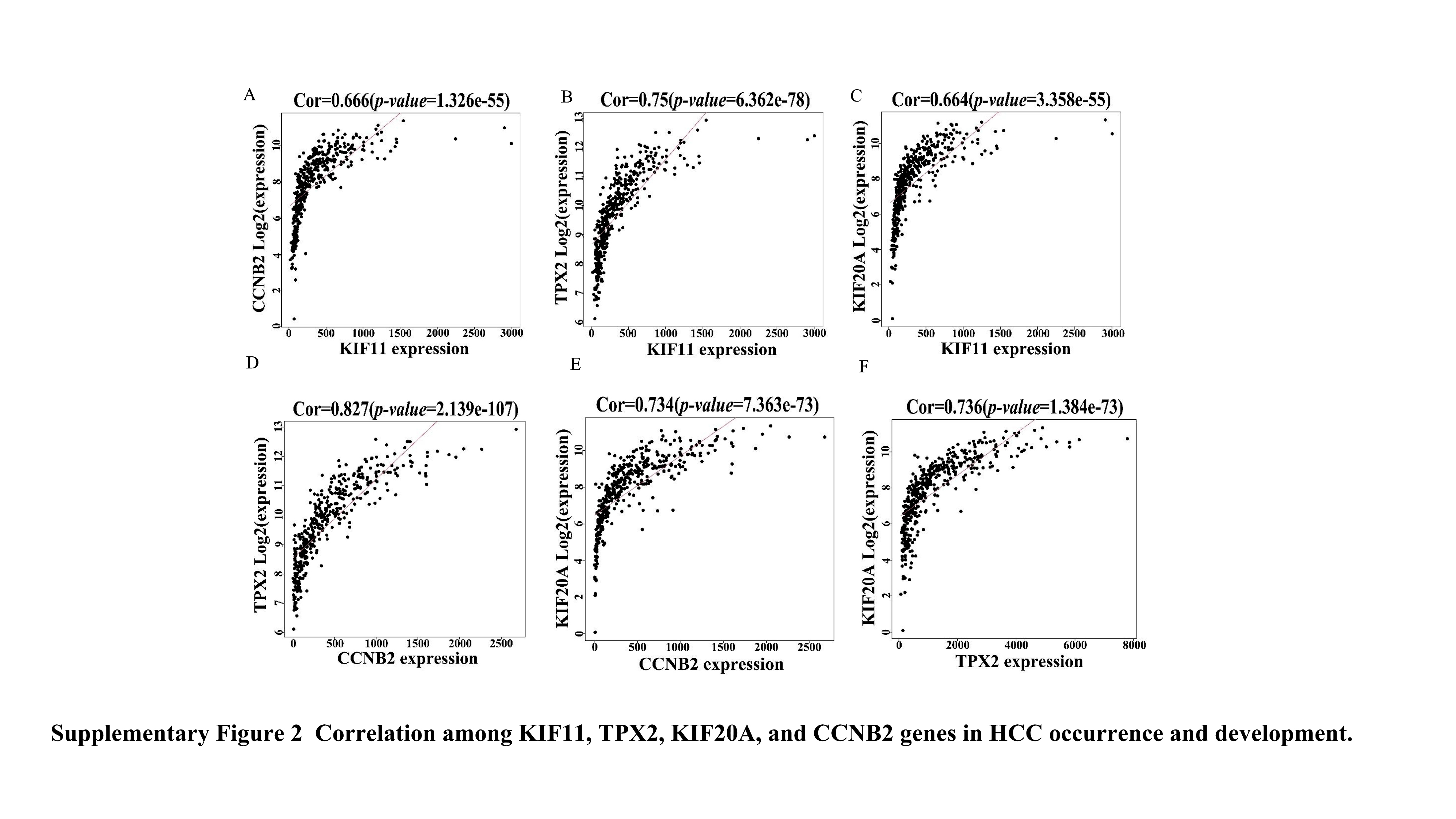

Supplement: FIGURE S2 — Correlation among KIF11, TPX2, KIF20A, and CCNB2 genes in HCC occurrence and development. Correlation analysis of KIF11, TPX2, KIF20A, and CCNB2 in HCC occurrence and development showed the potential relationships among these independent prognostic genes. Correlation coefficient (Cor) represents the correlation coefficient. p-value < 0.05 indicates a statistically significant difference. [file Image_2.JPEG]
