# Supplementary material for: Screening and Identification of Potential Biomarkers in Hepatitis B Virus-Related Hepatocellular Carcinoma by Bioinformatics Analysis
Source: Front Genet. 2020 Sep 30;11:555537. doi: 10.3389/fgene.2020.555537 (PMC7556301; doi:10.3389/fgene.2020.555537)
Supplement: TABLE S1 — The Top 20 up-regulated and 20 down-regulated DEGs between HCC and Normal tissues. [file Table_1.pdf]

Supplementary Table 1 The Top 20 up-regulated and 20 down-regulated DEGs between HCC and Normal tissues.

Gene	log2(FC)	*p-value*	FDR	State
PLVAP	2.922283	1.79E-64	1.79E-61	Upregulated
UBE2T	3.215664	1.40E-56	1.02E-53	Upregulated
CDKN3	3.913054	7.20E-54	4.26E-51	Upregulated
CDCA5	3.44238	1.07E-50	5.09E-48	Upregulated
CENPF	3.90067	1.89E-50	8.58E-48	Upregulated
PRC1	3.238714	1.49E-48	5.42E-46	Upregulated
CCNB2	3.733412	2.98E-48	1.07E-45	Upregulated
KIF4A	3.929729	3.29E-48	1.15E-45	Upregulated
CCNB1	3.282826	1.87E-47	6.02E-45	Upregulated
NDC80	3.318388	2.44E-47	7.69E-45	Upregulated
COL15A1	3.808312	6.33E-47	1.93E-44	Upregulated
CD34	2.103339	1.65E-46	4.61E-44	Upregulated
ASPM	3.737936	2.91E-46	7.89E-44	Upregulated
PTTG1	3.729616	1.37E-45	3.58E-43	Upregulated
CDK1	3.49655	2.92E-45	7.56E-43	Upregulated
KIF20A	3.725887	5.65E-45	1.39E-42	Upregulated
CDC20	4.275474	1.34E-44	3.08E-42	Upregulated
TOP2A	3.922546	2.60E-44	5.93E-42	Upregulated
PLK1	3.55609	3.31E-42	6.45E-40	Upregulated
FLVCR1	2.208538	5.64E-42	1.08E-39	Upregulated

Gene	log2(FC)	*p-value*	FDR	State
ADAMTS1	-2.82269	9.17E-129	2.66E-124	Downregulated
STAB2	-4.85756	1.82E-112	2.31E-108	Downregulated
OIT3	-3.25345	2.39E-112	2.31E-108	Downregulated
CFP	-3.40972	8.00E-95	4.64E-91	Downregulated
ECM1	-3.06658	5.86E-92	2.83E-88	Downregulated
BMPER	-4.50854	6.91E-90	2.51E-86	Downregulated
ANGPTL6	-2.97083	4.47E-88	1.44E-84	Downregulated
CLEC4G	-5.40445	9.11E-83	2.40E-79	Downregulated
CRHBP	-4.4779	1.64E-78	3.96E-75	Downregulated
PTH1R	-3.24519	5.70E-75	1.18E-71	Downregulated
MARCO	-4.85791	2.54E-74	4.61E-71	Downregulated
VIPR1	-3.47018	4.88E-74	8.32E-71	Downregulated
FCN3	-4.09928	1.56E-72	2.51E-69	Downregulated
FCN2	-4.89028	2.47E-72	3.76E-69	Downregulated
COLEC10	-3.9449	7.99E-66	9.26E-63	Downregulated
CETP	-2.82036	8.36E-65	8.97E-62	Downregulated
LIFR	-2.81766	1.10E-63	1.06E-60	Downregulated
AADAT	-2.78683	4.53E-60	4.10E-57	Downregulated
CLEC1B	-5.21772	9.53E-58	7.47E-55	Downregulated
PZP	-4.32388	2.31E-57	1.77E-54	Downregulated
