# Supplementary material for: Screening and Identification of Potential Biomarkers in Hepatitis B Virus-Related Hepatocellular Carcinoma by Bioinformatics Analysis
Source: Front Genet. 2020 Sep 30;11:555537. doi: 10.3389/fgene.2020.555537 (PMC7556301; doi:10.3389/fgene.2020.555537)
Supplement: TABLE S2 — The Top 15 significantly enriched GO terms of the 103 up-regulated DEGs. [file Table_2.pdf]

Supplementary Table 2 The Top 15 significantly enriched GO terms of the 103 up-regulated DEGs.

GO	ID	Description	Ratio	FDR	Gene	Count
MF	GO:0019901	protein kinase binding	0.115789474	0.009375	CCNB1/FAM83D/E2F1/CCNE2/KIF11/PRC1/CDKN2C/PLK1/TPX2/CCNA2/KIF20A	11
MF	GO:0019899	enzyme binding	0.094736842	0.036291	RFC4/POLD1/SORT1/SPDL1/CDC20/MCM2/PLCB1/HMGA1/TOP2A	9
MF	GO:0003682	chromatin binding	0.031578947	0.04391	CDK1/TOP2A/UBE2T	3
MF	GO:0048037	cofactor binding	0.031578947	0.047883	TYMS/POLD1/TP53I3	3
MF	GO:0008022	protein C-terminus binding	0.031578947	0.004771	CDC20/TOP2A/MAD2L1	3
CC	GO:0005819	spindle	0.21	7.08E-15	CENPF/KIF20A/KIF4A/ASPM/BUB1B/CDC20/PRC1/CDK1/CDC6/ECT2/PLK1/KIF11/CCNB1/CAPG/TPX2/CKAP2/FAM83D/MAD2L1/RAB11FIP4/SPDL1/INCENP	21
CC	GO:0098687	chromosomal region	0.17	1.66E-10	CENPF/BUB1B/NDC80/CDK1/CDCA5/PLK1/CCNB1/ZWINT/RECQL4/MTBP/MCM2/DSCC1/CENPU/MAD2L1/SPDL1/POLD1/INCENP	17
CC	GO:0000922	spindle pole	0.13	1.51E-10	CENPF/ASPM/CDC20/PRC1/CDC6/PLK1/KIF11/CCNB1/TPX2/CKAP2/FAM83D/MAD2L1/SPDL1	13
CC	GO:0000775	chromosome, centromeric region	0.13	7.41E-10	CENPF/BUB1B/NDC80/CDCA5/PLK1/CCNB1/ZWINT/MTBP/DSCC1/CENPU/MAD2L1/SPDL1/INCENP	13
CC	GO:0000793	condensed chromosome	0.12	1.51E-08	CENPF/BUB1B/NDC80/TOP2A/CDCA5/PLK1/CCNB1/ZWINT/CENPU/MAD2L1/SPDL1/INCENP	12
BP	GO:0000280	nuclear division	0.234693878	1.62E-15	KIF4A/ASPM/MYBL2/BUB1B/CDC20/NDC80/PRC1/TOP2A/CDC6/CDCA5/PLK1/NUSAP1/KIF11/CCNB1/ZWINT/MTBP/DSCC1/TPX2/MAD2L1/PLCB1/SPDL1/INCENP	23
BP	GO:0048285	organelle fission	0.234693878	8.30E-15	KIF4A/ASPM/MYBL2/BUB1B/CDC20/NDC80/PRC1/TOP2A/CDC6/CDCA5/PLK1/NUSAP1/KIF11/CCNB1/ZWINT/MTBP/DSCC1/TPX2/MAD2L1/PLCB1/SPDL1/INCENP	23
BP	GO:0140014	mitotic nuclear division	0.204081633	1.62E-15	CENPF/KIF4A/MYBL2/BUB1B/CDC20/NDC80/PRC1/CDC6/CDCA5/PLK1/NUSAP1/KIF11/CCNB1/ZWINT/MTBP/DSCC1/TPX2/MAD2L1/SPDL1/INCENP	20
BP	GO:0007059	chromosome segregation	0.193877551	1.51E-13	CENPF/KIF4A/BUB1B/CDC20/NDC80/PRC1/TOP2A/CDC6/ECT2/CDCA5/PLK1/NUSAP1/CCNB1/ZWINT/DSCC1/FAM83D/MAD2L1/SPDL1/INCENP	19
BP	GO:1901987	regulation of cell cycle phase transition	0.193877551	6.38E-10	CENPF/BUB1B/GTSE1/CDC20/NDC80/CDK1/CDC6/E2F1/CDCA5/PLK1/CCNB1/MTBP/TPX2/CDKN2C/FAM83D/MAD2L1/ARID3A/PLCB1/SPDL1	19
