# Supplementary material for: Screening and Identification of Potential Biomarkers in Hepatitis B Virus-Related Hepatocellular Carcinoma by Bioinformatics Analysis
Source: Front Genet. 2020 Sep 30;11:555537. doi: 10.3389/fgene.2020.555537 (PMC7556301; doi:10.3389/fgene.2020.555537)
Supplement: TABLE S3 — Enriched KEGG pathyways of the 103 up-regulated DEGs. [file Table_3.pdf]

Supplementary Table 3 Enriched KEGG pathways of the 103 up-regulated DEGS.

ID	Description	FDR	Gene	Count
hsa04110	Cell cycle	2.77E-12	CCNB2/BUB1B/CDC20/CDK1/PTTG1/CDC6/E2F1/CCNA2/PLK1/CCNB1/MCM2/CCNE2/CDKN2C/MAD2L	14
hsa04115	p53 signaling pathway	2.28E-05	CCNB2/GTSE1/CDK1/RRM2/CCNB1/CCNE2/TP53I3	7
hsa04114	Oocyte meiosis	6.27E-05	CCNB2/CDC20/CDK1/PTTG1/PLK1/CCNB1/CCNE2/MAD2L	8
hsa05166	Human T-cell leukemia virus 1 infection	0.000314	CCNB2/BUB1B/CDC20/PTTG1/E2F1/CCNA2/CCNE2/CDKN2C/MAD2L1	9
hsa04914	Progesterone-mediated oocyte maturation	0.001066	CCNB2/CDK1/CCNA2/PLK1/CCNB1/MAD2L1	6
hsa04218	Cellular senescence	0.001564	MYBL2/CCNB2/CDK1/E2F1/CCNA2/CCNB1/CCNE2	7
hsa03030	DNA replication	0.031735	MCM2/POLD1/RFC4	3
