# Supplementary material for: Screening and Identification of Potential Biomarkers in Hepatitis B Virus-Related Hepatocellular Carcinoma by Bioinformatics Analysis
Source: Front Genet. 2020 Sep 30;11:555537. doi: 10.3389/fgene.2020.555537 (PMC7556301; doi:10.3389/fgene.2020.555537)
Supplement: TABLE S4 — The Top 15 significantly enriched GO terms of the 127 down-regulated DEGs. [file Table_4.pdf]

Supplementary Table 4 The Top 15 significantly enriched GO terms of the 127 down-regulated DEGs.

GO	ID	Description	Ratio	FDR	Gene	Count
MF	GO:0048037	cofactor binding	0.14159292	1.56E-06	TAT/P3H2/KMO/PTGIS/ADH4/CYP2B6/STEAP4/CYP4A22/CYP4A11/ASPDH/CYP2C19/CYP39A1/AADAT/CYP1A2/IDO2/CYP2	16
MF	GO:0005506	iron ion binding	0.097345133	2.93E-07	P3H2/PTGIS/CYP2C8/CYP2B6/CYP4A22/CYP4A11/HBA2/CYP2C19/CYP39A1/CYP1A2/CYP26A1	11
MF	GO:0004497	monooxygenase activity	0.088495575	7.51E-08	KMO/PTGIS/CYP2C8/CYP2B6/CYP4A22/CYP4A11/CYP2C19/CYP39A1/CYP1A2/CYP26A1	10
MF	GO:0020037	heme binding	0.088495575	2.93E-07	PTGIS/CYP2B6/STEAP4/CYP4A22/CYP4A11/CYP2C19/CYP39A1/CYP1A2/IDO2/CYP26A1	10
MF	GO:0046906	tetrapyrrole binding	0.088495575	3.66E-07	PTGIS/CYP2B6/STEAP4/CYP4A22/CYP4A11/CYP2C19/CYP39A1/CYP1A2/IDO2/CYP26A1	10
CC	GO:0031012	extracellular matrix	0.091666667	0.006068491	P3H2/SRPX/DPT/DCN/ADAMTS13/ANGPTL6/CFP/ECM1/SFRP1/CCBE1/PZP	11
CC	GO:0062023	collagen-containing extracellular matrix	0.075	0.021987491	P3H2/SRPX/DPT/DCN/ANGPTL6/CFP/ECM/SFRP1/PZP	9
CC	GO:0005581	collagen trimer	0.058333333	0.000187285	C1QTNF1/DCN/COLEC10/CCBE1/FCN3/MARCO/FCN2	7
CC	GO:0072562	blood microparticle	0.041666667	0.04556051	HBA2/CD5L/PZP/FCN3/FCN2	5
CC	GO:0034358	plasma lipoprotein particle	0.033333333	0.003920237	LCAT/LPA/APOF/CETP	4
BP	GO:0044282	small molecule catabolic process	0.130434783	1.98E-05	TAT/KMO/ADH4/HK3/GSTZ1/CYP4A11/ASPA/AKR1D1/HAO2/CYP39A1/AADAT/LYVE1/IDO2/CYP26A1/STAB2	15
BP	GO:0016054	organic acid catabolic process	0.113043478	5.91E-06	TAT/KMO/GSTZ1/CYP4A11/ASPA/AKR1D1/HAO2/CYP39A1/AADAT/LYVE1/IDO2/CYP26A1/STAB2	13
BP	GO:0046395	carboxylic acid catabolic process	0.113043478	5.91E-06	TAT/KMO/GSTZ1/CYP4A11/ASPA/AKR1D1/HAO2/CYP39A1/AADAT/LYVE1/IDO2/CYP26A1/STAB2	13
BP	GO:1901615	organic hydroxy compound metabolic process	0.113043478	0.001570523	LCAT/IL1B/ADH4/RDH16/CYP4A11/RDH5/AKR1D1/APOF/HAND2/MOGAT2/CETP/CYP39A1/PTH1R	13
BP	GO:0008202	steroid metabolic process	0.104347826	0.000184728	LCAT/IL1B/CYP2C8/RDH16/CYP2B6/CYP2C19/RDH5/AKR1D1/APOF/CETP/CYP39A1/CYP1A2	12
