# Supplementary material for: Screening and Identification of Potential Biomarkers in Hepatitis B Virus-Related Hepatocellular Carcinoma by Bioinformatics Analysis
Source: Front Genet. 2020 Sep 30;11:555537. doi: 10.3389/fgene.2020.555537 (PMC7556301; doi:10.3389/fgene.2020.555537)
Supplement: TABLE S5 — Enriched KEGG pathyways of the 127 down-regulatde DEGs. [file Table_5.pdf]

Supplementary Table 5 Enriched KEGG pathways of the 127 down-regulated DEGs.

ID	Description	FDR	Gene	Count
hsa00830	Retinol metabolism	7.87E-08	CYP2C8/ADH4/RDH16/CYP2B6/CYP4A22/CYP4A11/UGT2B10/RDH5/CYP1A2/CYP26A1	10
hsa00590	Arachidonic acid metabolism	0.001948992	PTGIS/CYP2C8/CYP2B6/CYP4A22/CYP4A11/CYP2C19	6
hsa00982	Drug metabolism - cytochrome P450	0.002791279	CYP2C8/ADH4/CYP2B6/UGT2B10/CYP2C19/CYP1A2	6
hsa05204	Chemical carcinogenesis	0.004350501	CYP2C8/ADH4/UGT2B10/CYP2C19/CYP1A2/NAT2	6
hsa04978	Mineral absorption	0.006098904	MT1E/MT1G/MT1M/MT1F/MT1H	5
hsa00380	Tryptophan metabolism	0.015367676	KMO/AADAT/CYP1A2/IDO2	4
hsa00980	Metabolism of xenobiotics by cytochrome P450	0.015367676	AKR7A3/ADH4/CYP2B6/UGT2B10/CYP1A2	5
hsa00591	Linoleic acid metabolism	0.045288022	CYP2C8/CYP2C19/CYP1A2	3
