# Supplementary material for: Screening and Identification of Potential Biomarkers in Hepatitis B Virus-Related Hepatocellular Carcinoma by Bioinformatics Analysis
Source: Front Genet. 2020 Sep 30;11:555537. doi: 10.3389/fgene.2020.555537 (PMC7556301; doi:10.3389/fgene.2020.555537)
Supplement: TABLE S6 — Degree scores of the hub genes. [file Table_6.pdf]

Supplementary Table 6 Degree scores of the hub genes.

Rank	Gene	Score	log2(FC)	*p-value*	FDR	State
1	CDK1	51	3.49655	2.92E-45	7.56E-43	Upregulated
2	CCNB1	50	3.282826	1.87E-47	6.02E-45	Upregulated
2	CCNA2	50	3.517922	2.61E-35	2.72E-33	Upregulated
4	BUB1B	48	3.524702	8.47E-40	1.33E-37	Upregulated
4	CCNB2	48	3.733412	2.98E-48	1.07E-45	Upregulated
4	CDC20	48	4.275474	1.34E-44	3.08E-42	Upregulated
4	TOP2A	48	3.922546	2.60E-44	5.93E-42	Upregulated
8	KIF11	47	2.215634	9.96E-30	6.05E-28	Upregulated
8	PLK1	47	3.55609	3.31E-42	6.45E-40	Upregulated
10	MAD2L1	46	2.185421	3.39E-30	2.16E-28	Upregulated
10	KIF20A	46	3.725887	5.65E-45	1.39E-42	Upregulated
10	TPX2	46	2.124336	2.32E-24	8.42E-23	Upregulated
