# Supplementary material for: Screening and Identification of Potential Biomarkers in Hepatitis B Virus-Related Hepatocellular Carcinoma by Bioinformatics Analysis
Source: Front Genet. 2020 Sep 30;11:555537. doi: 10.3389/fgene.2020.555537 (PMC7556301; doi:10.3389/fgene.2020.555537)
Supplement: TABLE S7 — The Top 15 significantly enriched GO terms of the hub genes. [file Table_7.pdf]

Supplementary Table 7 The Top 15 significantly enriched GO terms of the hub genes.

GO	ID	Description	Ratio	FDR	Gene	Count
MF	GO:0008022	protein C-terminus binding	0.75	0.004771127	CDC20/TOP2A/MAD2L1	3
MF	GO:0004674	protein serine/threonine kinase activity	0.5	0.020434212	CDK1/BUB1B/PLK1	3
MF	GO:0042826	histone deacetylase binding	0.416666667	0.020434212	CDC20/TOP2A	2
MF	GO:0097472	cyclin-dependent protein kinase activity	0.416666667	0.001724091	CDK1/CCNA2	2
MF	GO:0019901	protein kinase binding	0.416666667	0.0093746	CCNB1/KIF11/PLK1/TPX2/CCNA2/KIF20A	5
CC	GO:0005819	spindle	0.416666667	1.42E-12	CDK1/CCNB1/BUB1B/CDC20/KIF11/PLK1/MAD2L1/KIF20A/TPX2	9
CC	GO:0000922	spindle pole	0.25	5.84E-09	CCNB1/CDC20/KIF11/PLK1/MAD2L1/TPX2	6
CC	GO:0000793	condensed chromosome	0.25	1.22E-06	CCNB1/BUB1B/TOP2A/PLK1/MAD2L1	5
CC	GO:0098687	chromosomal region	0.166666667	6.62E-06	CDK1/CCNB1/BUB1B/PLK1/MAD2L1	5
CC	GO:0005813	centrosome	0.166666667	2.68E-05	CDK1/CCNB1/CCNB2/CDC20/PLK1	5
BP	GO:0000280	nuclear division	0.666666667	2.62E-09	CCNB1/BUB1B/CDC20/TOP2A/KIF11/PLK1/MAD2L1/TPX2	8
BP	GO:0048285	organelle fission	0.666666667	3.92E-09	CCNB1/BUB1B/CDC20/TOP2A/KIF11/PLK1/MAD2L1/TPX2	8
BP	GO:0000226	microtubule cytoskeleton organization	0.666666667	4.33E-09	CDK1/CCNB1/CDC20/KIF11/PLK1/MAD2L1/KIF20A/TPX2	8
BP	GO:0140014	mitotic nuclear division	0.583333333	4.33E-09	CCNB1/BUB1B/CDC20/KIF11/PLK1/MAD2L1/TPX2	7
BP	GO:1901990	regulation of mitotic cell cycle phase transition	0.583333333	5.51E-08	CDK1/CCNB1/BUB1B/CDC20/PLK1/MAD2L1/TPX2	7
