# Supplementary material for: Screening and Identification of Potential Biomarkers in Hepatitis B Virus-Related Hepatocellular Carcinoma by Bioinformatics Analysis
Source: Front Genet. 2020 Sep 30;11:555537. doi: 10.3389/fgene.2020.555537 (PMC7556301; doi:10.3389/fgene.2020.555537)
Supplement: TABLE S8 — Enriched KEGG pathyways of the hub genes. [file Table_8.pdf]

**Supplementary Table 8 Enriched KEGG pathways of the hub genes.**

| ID | Description | FDR | Gene | Count |
|---|---|---|---|---|
| hsa04110 | Cell cycle | 4.48E-13 | CDK1/CCNB1/CCNA2/BUB1B/CCNB2/CDC20PLK1/MAD2L1 | 8 |
| hsa04914 | Progesterone-mediated oocyte maturation | 2.36E-09 | CDK1/CCNB1/CCNA2/CCNB2/PLK1/MAD2L1 | 6 |
| hsa04114 | Oocyte meiosis | 7.53E-09 | CDK1/CCNB1/CCNB2/CDC20/PLK1/MAD2L1 | 6 |
| hsa05166 | Human T-cell leukemia virus 1 infection | 7.87E-06 | CCNA2/BUB1B/CCNB2/CDC20/MAD2L1 | 5 |
| hsa04218 | Cellular senescence | 6.64E-05 | CDK1/CCNB1/CCNA2/CCNB2 | 4 |
| hsa04115 | p53 signaling pathway | 0.000172898 | CDK1/CCNB1/CCNB2 | 3 |
| hsa04068 | FoxO signaling pathway | 0.000879728 | CCNB1/CCNB2/PLK1 | 3 |
| hsa05203 | Viral carcinogenesis | 0.002692946 | CDK1/CCNA2/CDC20 | 3 |
| hsa05170 | Human immunodeficiency virus 1 infection | 0.002793135 | CDK1/CCNB1/CCNB2 | 3 |
